# Rescue Paracetamol in Postoperative Pain Management in Extremely Low Birth Weight Neonates Following Abdominal Surgery: A Single Unit Retrospective Study

**DOI:** 10.3389/fped.2022.895040

**Published:** 2022-06-23

**Authors:** Hana Cihlarova, Lenka Bencova, Blanka Zlatohlavkova, Karel Allegaert, Pavla Pokorna

**Affiliations:** ^1^Division of Neonatology, Clinic of Gynaecology and Obstetrics, First Faculty of Medicine, Charles University and General University Hospital, Prague, Czechia; ^2^Institute of Pharmacology, First Faculty of Medicine, Charles University and General University Hospital, Prague, Czechia; ^3^Department of Hospital Pharmacy, Erasmus MC, Rotterdam, Netherlands; ^4^Department of Pharmaceutical and Pharmacological Sciences, KU Leuven, Leuven, Belgium; ^5^Department of Development and Regeneration, KU Leuven, Leuven, Belgium; ^6^Department of Paediatrics and Inherited Metabolic Disorders, First Faculty of Medicine, Charles University and General University Hospital, Prague, Czechia; ^7^Department of Physiology and Pharmacology, Karolinska Institutet and Karolinska University Hospital, Stockholm, Sweden; ^8^Intensive Care and Department of Paediatric Surgery, Erasmus Medical Centre Sophia Children's Hospital, Rotterdam, Netherlands

**Keywords:** extremely low birth weight neonates, postoperative pain, COMFORTneo score, paracetamol, opioid consumption

## Abstract

**Background:**

Intravenous paracetamol added to morphine reduces postoperative morphine consumption in (near)term neonates. However, there are only sparse data on intravenous paracetamol as multimodal strategy in extremely low birth weight (ELBW) neonates.

**Objectives:**

This study aims to assess the effects of rescue intravenous paracetamol on postoperative pain management (≤48 h postoperatively) in relation to both analgesic efficacy (validated pain assessment, drug consumption, adequate rescue medication) and safety (hypotension and bradycardia). This rescue practice was part of a standardized pain management approach in a single neonatal intensive care unit (NICU).

**Methods:**

A single-center retrospective observational study included 20 ELBW neonates, who underwent major abdominal surgery. The primary endpoints of the postoperative study period were pain intensity, over-sedation, time to first rescue analgesic dose, and the effect of paracetamol on opiate consumption. Secondary endpoints were safety parameters (hypotension, bradycardia). And as tertiary endpoints, the determinants of long-term outcome were evaluated (i.e., duration of mechanical ventilation, intraventricular hemorrhage - IVH, periventricular leukomalacia - PVL, postnatal growth restriction, stage of chronic lung disease – CLD or neurodevelopmental outcome according to Bayley-II Scales of Infant Development at 18–24 months).

**Results:**

All neonates received continuous opioids (sufentanil or morphine) and 13/20 also intravenous paracetamol as rescue pain medication during a 48-h postoperative period. Although opioid consumption was equal in the non-paracetamol and the paracetamol group over 48 h, the non-paracetamol group was characterized by oversedation (COMFORTneo < 9), a higher incidence of severe hypotension, and younger postnatal age (*p* < 0.05). All long-term outcome findings were similar between both groups.

**Conclusions:**

Our study focused on postoperative pain management in ELBW neonates, and showed that intravenous paracetamol seems to be safe. Prospective validation of dosage regimens of analgesic drugs is needed to achieve efficacy goals.

## Introduction

In 2016, a multidisciplinary ESPNIC (The European Society of Pediatric and Neonatal Intensive Care) position statement was published guiding professionals in assessing and re-evaluating treatment interventions for pain, distress, inadequate sedation, withdrawal syndrome, and delirium in the pediatric populations for various pain profiles across ages (1, 2). It is widely accepted that neonates can experience pain, and knowledge of the relevance of pain management has increased significantly over the decades (3–5). However, short-term and long-term consequences of pain management approaches remain sparse in extremely low birth weight (ELBW) infants (6–10).

Unfortunately, the treatment of ELBW neonates is still difficult at present, and setting the key endpoints in neonatal analgesic clinical trials is necessary (11, 12). First, validated pain assessment tools are crucial for targeted analgesia. However, out of 65 scores, only 37% were validated for premature neonates, and only one score (the Pain Assessment Tool) for postoperative pain in extremely premature neonates (13). Second, age-appropriate drugs and dose adjustments of analgesic drugs used to adequately treat pain are also important while a still high percentage of analgesic drugs is used in an unlicensed or off-label manner in the intensive care unit (NICU) settings (14). For postoperative pain, in neonates aged between 36 weeks gestational age (GA) and infants <1-year-old, an intermittent intravenous paracetamol dose of 10 mg/kg per 6 h resulted in a significant reduction in opioid use and exposure following non-cardiac major surgery (15).

This is, even more, the case in the specific setting of pain management in ELBW for necrotizing enterocolitis or abdominal surgery, as recently observed by ten Barge et al. (16). In their dataset on 79 preterm cases with necrotizing enterocolitis, the authors concluded that the majority experienced pain, and in some cases, this pain persisted for several hours. Based on a similar concept of using data collected during clinical care within one neonatal intensive care unit (NICU), we performed a comparative analysis and audit of postoperative pain management in ELBW neonates after abdominal surgery.

Consequently, this study aimed to evaluate the unit protocol for postoperative pain management (≤48 h) to achieve postoperative analgesic efficacy goals (e.g., validated pain scores) after major abdominal surgery in ELBW infants. This standardized local approach included the use of single-dose paracetamol as a rescue drug. We hereby documented drug utilization, effective drug dosing (pain scores within the target zone, with emphasis on rescue intravenous paracetamol) as well as safety parameters (e.g., hypotension and/or bradycardia) related to the use of analgesics and sedatives in ELBW neonates.

## Methods

### Setting and Study Population

The retrospective single unit study included ELBW neonates admitted to the Level III NICU of General University Hospital, 1st Faculty of Medicine of the Charles University in Prague, Czech Republic, who underwent acute major abdominal surgery between January 2014 and December 2019. An institutional review board (IRB) approval for publication of the study was obtained (No. 117 248/21 S-IV). The use of anonymous data for scientific purposes is part of general informed consent, which parents signed during admission to the hospital.

Inclusion criteria were birth weight (BW) <1,000 g and gestational age (GA) ≤28 weeks; abdominal surgery [e.g., laparotomy due to necrotizing enterocolitis (NEC), spontaneous intestinal perforation (SIP), bowel obstruction or volvulus] by postmenstrual age (PMA) ≤36 weeks.

Exclusion criteria were the refusal of an informed consent form. One of the operated patients was excluded for infaust prognosis (pan intestinal NEC) and decision of care termination at the time of surgery. Furthermore, we did not enroll the same patients undergoing the second planned operation (stoma closure), because the planned operations and the first days of postoperative care took place in another center.

Enrolled neonates were stratified into two groups according to paracetamol administration as rescue analgesic therapy: a paracetamol (P) group, *n* = 13; and a non-paracetamol (non-P) group, *n* = 7. In all cases, a treatment period until 48 h post-surgery was assessed.

### Data Collection

Demographic characteristics were collected: weeks of gestational age (GA), postmenstrual age (PMA) at surgery, birth weight (BW) and the actual body weight at surgery (g), gender (female/male), Apgar score, and diagnosis (indication for surgery).

The primary endpoints parameters were pain intensity, over-sedation, time to first rescue analgesic dose, and the effect of paracetamol on opiate consumption. Safety parameters (bradycardia <80/min; hypotension defined as a mean blood pressure of <10^th^ percentile-a short episode without treatment or an episode treated with catecholamines) were the secondary endpoints of the study.

As long-term outcome parameters (tertiary endpoints) were evaluated mechanical ventilation duration, length of hospital stay, grade of intraventricular hemorrhage (IVH) and periventricular leukomalacia (PVL), stage of chronic lung disease (CLD); postnatal growth restriction (body weight and height <10^th^ centile according to Fenton growth chart) and breastfeeding on hospital discharge; and long-term neurodevelopmental outcomes evaluated by Bayley Scales of Infant Development-BSID-II-mental (MDI) and psychomotor (PDI) developmental index-standardized in infants (cut-off values for the definition of moderate-severe neurodevelopmental delay impairment of MDI and PDI < 70).

### Pain Assessment

Based on the pre-existing unit protocol for pain management, nurses assessed each neonate while resting using a COMFORTneo score at least four times per day. Additionally, the nurses monitored episodes of “obvious pain” (yes/no). The obvious pain score is a locally adapted and internally validated score that simplifies a subjective evaluation system such as the Numeric Rating Scale (NRS) for moderate and severe pain (NRS ≥ 4). Obvious pain assessment is a part of the nurses' daily documentation evaluated at least once every 3–6 h. COMFORTneo is a validated pain score even for very premature neonates, consisting of 6 behavioral items (alertness, calmness/agitation; crying/breathing reaction in ventilated patients; movements; muscle tone; and facial tension) (17). In our unit protocol, a target score range of 9–14 was used. A score of 14 is the cut-off value at which some non-pharmacological interventions were used to reduce discomfort (e.g., positioning, non-nutritive sucking) before increasing medication. The COMFORTneo scores below 9 in sedated neonates suggest over-sedation.

### Medication Utilization

Analgesic drug dosages were based on standardized international guidelines (5, 18–20). All enrolled patients were treated with opioids (morphine or sufentanil) preoperatively because of severe abdominal disease. Moreover, opioids were given also as part of a combined general anesthesia protocol. Operative drug doses were not included in the analysis. In the postoperative period, along with continuous opioids titrated to the desired postoperative effects, paracetamol and other analgesic drugs (opioids boluses, ketamine, propofol) were administered as rescue medications.

The indication for the rescue paracetamol administration was either one event with a COMFORTneo > 14 or one observation of obvious pain, or both.

#### Paracetamol

Paracetamol (Paracetamol Kabi inj, 10 mg/1 mL, Fresenius Kabi s. r. o., Prague, Czech Republic) was administrated intravenously (dose 7.5 mg/kg as a single dose or every 6–8 h over 15 min). The loading dose of paracetamol was not administered at that period.

#### Opioids

*Morphine* **(**Morphin Biotika 1 % inj., 10 000 μg/mL, BB Pharma a. s., Prague, Czech Republic) given an initial bolus (10–40 μg/kg/ over 10 min) followed by a continuous intravenous infusion (2.5–10 μg/kg/h, a maximum dose of 20 μg/kg/h in ventilated neonates).

*Sufentanil* (Sufentanil Torrex 5 μg/mL inj., Chiesi Pharmaceuticals GmbH, Vienna, Austria), an initial bolus of 0.2 μg/kg administered for 10 min intravenously followed by a continuous infusion of 0.05-0.2 μg/kg/h. Sufentanil average daily dose was converted to morphine equivalents (IV sufentanil 0.1 mg = IV morphine 100 mg) (21, 22).

#### Other Drugs

*Ketamine* (Calypsol inj 50 mg/mL inj., Gedeon Richter Plc., Budapest, Hungary), given in a single dose (2–3 mg/kg).

*Propofol* (Propofol MCT/LCT Fresenius 10 mg/mL inj., Fresenius Kabi Deutschland GmbH, Hamburg, Germany), given in a single dose (2 mg/kg).

### Statistical Analysis

Basic features were summarized by descriptive statistics such as median, interquartile range (IQR), or range of variables. Mann–Whitney (U-test) or Fisher's exact test was used to comparing patients exposed to paracetamol (P-group) to those without paracetamol (non-P group) exposure. The results are reported in the form of the median (IQR).

## Results

### Study Population

Of the 1,277 NICU admissions during the study period 2014-2019 about 417 patients were ELBW neonates (birth weight < 1,000 g). Forty eight suffered from NEC (Modified Bell Criteria, stage ≥II), and 8 neonates were diagnosed with SIP (pneumoperitoneum on X-ray). Surgical treatment was indicated in 17 patients with NEC, 4 patients with SIP, and 1 patient with volvulus.

The characteristics of the studied population are shown in Table 1. Twenty ELBW neonates (11 females and 9 males), who underwent laparotomy between 2014 and 2019 were enrolled in the study. The median (IQR) birth weight was 667 (558–749) g, and the median (IQR) gestational age (GA) was 24 weeks and 5 days (24^+1^ - 25^+2^). A statistically significant difference between the P and non-P groups was found in median PMA at the time of surgery, in the P group at 28 weeks and 2 days (27+0 - 30+0) vs. non-P group at 26 weeks and 3 days (26+2−26+6), thus 29 vs. 8 days of postnatal age, respectively, (*p* = 0.034). The most frequent surgical diagnosis was necrotizing enterocolitis (NEC) in 15 cases, spontaneous intestinal perforation (SIP) was diagnosed in 4 cases, and bowel obstruction in 1 patient, none of the included patients died during the study period.

**Table 1:** Study population and outcome parameters.

**Parameter**	**All patients** **(*n* = 20)**	**P group** **(*n* = 13)**	**Non-P group** **(*n* = 7)**	***p-*value**
Birth weight (g)^e^	667 (558–749)	660 (585–735)	675 (585–735)	0.178
Gender female/male	11/9	8/5	6/1	-
GA (weeks^+days^)^e^	24^+5^ (24^+1^-25^+2^)	24^+2^ (24^+0^-25^+2^)	25^+2^ (24^+4^-25^+6^)	0.121
Apgar 1^e^	3.5 (3–6.25)	5 (3–7)	3 (2.5–3.5)	0.207
Apgar 5^e^	7 (6–8)	7 (6–8)	6 (5.5–7)	0.352
Apgar 10^e^	8 (8–9)	9 (8–9)	8 (7.5–8)	0.160
PMA (weeks^+days^)^e^	27^+0^ (26^+3^-28^+4^)	28^+2^ (27^+0^-30^+0^)	26^+3^ (26^+2^-26^+6^)	**0.034**
Weight at operation (g)	730 (638–850)	745 (670–900)	650 (540–807.5)	0.178
Diagnosis NEC/SIP/bowel obstruction	*n* = 15/4/1	*n* = 10/2/1	*n* = 5/2/-	NA
Length of mechanical ventilation (days)^e^	33 (20–41)	35 (29.3–39.5)	22 (17.3–42.8)	0.383
Length of stay (days)^e^	126.5 (109–134)	126 (110–135.8)	127 (105.3–137.5)	0.202
Bradycardia < 80/min	*n* = 2	*n* = 2	*n* = 0	NA
IVH^e^ (grade)	1 (0–2)	1 (0–1)	2 (0–2)	0.237
PVL^e^ (grade)	0 (0–0)	0 (0–0)	0 (0–0)	0.201
PDA^e^ (severity)	1 (0–1)	1 (0–1)	1 (1–1)	0.295*
Hypotension^e^ (severity)	1 (0–2)	1 (0–1)	2 (2–2)	0.012*
MDI/PDI^e^ (*n* = 16)	85 (51–91)/ 79 (50–95)	83.5 (50–90)/ 72.5 (50–94)	87.5 (50–99)/ 85 (67–96)	0.855/ 0.504

*P, paracetamol rescue;*

e median (interquartile range), NA, not applicable; NEC, necrotizing enterocolitis; SIP, spontaneous intestinal perforation; IVH, intraventricular hemorrhage; PVL, periventricular leukomalacia; PDA, persistent ductus arteriosus (0 = none, 1 = spontaneous closure, 2 = pharmacological treatment, 3 = surgery), hypotension (0 = none, 1 = bellow 10th percentile, 2=catecholamines), MDI/PDI mental/psychomotor developmental index according to Bayley II, PMA postmenstrual age, GA, gestational age; statistical testing was provided by U-test or (^*^) Fisher's exact test. The bold values are statistically significant values (p < 0.05).

### Primary Endpoints

#### Pain Assessment

All enrolled patients were scored with a COMFORTneo scale. In addition, the nurses reported “obvious pain,” when the discomfort was noted. The nurses performed approximately five COMFORTneo assessments per day on each patient. During the 48-h follow-up period, 57% of the measured COMFORTneo scores were in the target range (9–14), which corresponds to 27 h of adequate pain control in the follow-up period. 23.5% of scores (12 h) corresponded to over-sedation and, conversely, insufficient pain control was reported by 12% of scores (6 h) (Table 2).

**Table 2:** Primary endpoints: COMFORTneo pain assessment in treatment groups within 48 h after surgery.

**COMFORTneo** **scores**	**All patients** **(%)**	**P group** **(%)**	**Non-P group** **(%)**	***p-value***
Time proportion in the target range (9–14)^e^	56.5 (47.5–62)	57 (44.7–62.2)	56 (51.5–6.5)	0.905
Time proportion out of the range^e^	43.5 (38–52.5)	43 (37.8–55.3)	44 (38.5–48.5)	0.905
Bellow target (<9)^e^	23.5 (0–39)	11 (0–31.3)	36 (27–43)	**0.027**
Above target (>14)^e^	12 (0–43.5)	33 (0–51.3)	8 (0–12.5)	0.190
	**All patients** **(*****n*****)**	**P group** **(*****n*****)**	**Non-P group** **(*****n*****)**	
COMFORTneo number of assessments per patient per day^e^	4.9	4.5	4.9	0.936

*P, paracetamol rescue;*

e *median (interquartile range); all statistical testing was provided by U-test.*

*The bold values are statistically significant values (p < 0.05)*.

The non-P group had lower scores than the P group, significantly during the first 24 h after surgery (*p* = 0.015, U-test) (Supplementary Figure S1). The score of the non-P group (median 8) signalized over-sedation, while the P group was in the target range (median 13). A score shift was observed on the second postoperative day (24–48 h) when the non-P group achieved the target range, and the P group was not under adequate pain control with a median score of 15 (11-15.3). More events of obvious pain were also reported by nurses in the P group, but the difference was not statistically significant, as detailed in Table 3. Therefore, the bolus rescue doses (RD) of any analgesics were administered more frequently in the P group (*p* < 0.017, U-test). Intravenous paracetamol RD was indicated if COMFORTneo exceeded 14 (*n* = 1), when obvious pain was observed (*n* = 6), or both (*n* = 4). In 2 patients, paracetamol was added immediately after surgery without documented increased pain score. Accordingly, paracetamol RD was administrated in 10/13 cases although the median (IQR) COMFORTneo score was 12 (9-13.3), i.e. within the target.

**Table 3:** Primary endpoints: medication and pain control in treatment groups for three periods within 48 h after surgery.

**Parameter**	**0–12 h**				**12–24 h**				**24-48 h**			
	**All** **(*n* = 20)**	**P group** **(*n* = 13)**	**Non-P group** **(*n* = 7)**	***p-*value**	**All** **(*n* = 20)**	**P group**	**Non-P group**	***p-*value**	**All** **(*n* = 20)**	**P group**	**Non-P group**	***p-*value**
Total number of drugs per patient^e^	1	2 (max. 4)	1 (max.1)	**0.003**	1.5	2 (max. 4)	1 (max. 1)	**0.017**	2	2 (max. 3)	1 (max. 2)	0.683
Opiate EqCD μg/kg per patient (n=20)^e^	2900 (186–3,950)	3350 (195–4,050)	334 (134–3,475)	0.234	2105 (150–3,685)	2870 (231–3,990)	120 (114–3,188)	0.178	570 (248–6,975)	585 (270–7,488)	376 (204–4,200)	0.476
	**0–24 h**								**24–48 h**			
	**All** **(*****n*** **=** **20)**	**P group** **(*****n*** **=** **13)**			**Non-P group** **(*****n*** **=** **7)**			***p***	**All** **(*****n*** **=** **20)**	**P group**	**Non-P group**	***p-*** **value**
COMFORTneo score per patient^e^	9.5 (8–13)	13 (9–13.3)			8 (7–8.8)			**0.015**	12.5 (11–15)	15 (11–15.3)	12 (10.3–12)	0.161
Number of “obvious pain” episodes per patient per day^e^	2 (2.4)	2.5 (2.7)			1 (1.3)			0.221	1.7 (1.9)	2.1 (1.9)	0.9 (1.6)	0.133

*P, paracetamol rescue;*

e *median (interquartile range), EqCD equivalent cumulative dose od opiates - cumulative dose of morphine and morphine equivalents (μg/kg); all statistical testing was provided by U-test.*

*The bold values are statistically significant values (p < 0.05)*.

The reassessment of the COMFORTneo was performed 5 (4–6) h after the RD because the patients were considered as comfortable and no obvious pain was reported. And the reassessment median score was insignificantly worse 13.9 (11-15) than the previous.

#### Medication Utilization

During the 48-h treatment period, all patients received continuous opioids, 13 patients (65%) received sufentanil, 12 patients (60%) morphine, 5 patients (25%) both sufentanil and morphine consecutively). Sixteen patients (80%) required an additional bolus analgesic drug to achieve sufficient pain control. The time to the first rescue dose was the median (IQR) 11.5 (3.5–24) h. Of these, 13 (81.3%) received paracetamol, 6 patients (37.5%) had ketamine, 4 patients (25%) received an additional opioid bolus and 1 patient (6.3%) propofol. The average analgesic daily dosage is shown in Table 4. The median for equivalent sufentanil and morphine doses (equivalent average daily dose of opiates - EqADD) did not differ between the groups (Supplementary Figure S2). The median paracetamol dose was 16 mg/kg/day in the cohort. According to the three postoperative periods (0–12, 12–24, and 24–48 h), the median number of drugs increased (1; 1,5; and 2 drugs, respectively) (Table 3).

**Table 4:** The average daily dose of analgesic drugs within 48 h after surgery.

**Parameter**	**All** **(*n* = 20)**	**P group** **(*n* = 13)**	**Non-P group** **(*n* = 7)**	***p-*value**
Time to first rescue analgesia (h)^e^	11.5 (3.5–24)	8 (3.8–19.8)	31 (10–31.8)	0.350
Paracetamol ADD mg/kg per patient^e^ (*n* = 13)	-	16 (7.4–25.4)	**-**	**-**
Sufentanil ADD μg/kg per patient^e^	6.1 (3–9.5) (*n* = 13/20)	7.7 (3–9.5) (*n* = 9/13)	4.9 (2–8) (n=4/7)	0.604
Morphine ADD μg/kg per patient^e^	243 (178–316) (*n* = 12/20)	280 (110–382) (*n* = 8/13)	227 (207–246) (*n* = 4/7)	0.683
Opiate EqADD μg/kg per patient^e^	3,085 (359–7,725)	3,105 (473–8,209)	403 (220–5,431)	0.178

*P, paracetamol rescue;*

e *median (interquartile range); ADD, average daily dose; EqADD, equivalent average daily dose of opiates - average daily dose of morphine and morphine equivalents (μg/kg); all statistical testing was provided by U-test*.

### Secondary and Tertiary Endpoints

Safety parameters such as bradycardia (<80/min) event were observed in 2 patients in the P group (10%), while no severe bradycardia was documented in the non-P group patients. In contrast, events of serious hypotension (treated with catecholamines) were more commonly documented in the non-P group (*p* = 0.012, Fisher's test) (Table 1).

The tertiary endpoints are shown also in Table 1. All determinants, such as length of mechanical ventilation, length of hospital stay, grades of IVH and PVL, stage of CLD, postnatal growth restriction, or breastfeeding on hospital discharge were not statistically different between groups. The long-term neurodevelopmental outcome according to the Bayley Scales of Infant Development-BSID-II mental (MDI) and psychomotor (PDI) developmental indexes standardized in infants aged 18–24 months has so far been evaluated in 16 (80%) of former ELWB neonates in the cohort. There was no difference between groups in the Bayley MDI and PDI developmental scales.

## Discussion

The main goal of this retrospective study was to generate additional data on the efficacy of paracetamol (effects to reduce postoperative pain) and its safety in ELBW neonates following major abdominal surgery. In this specific setting, and taking the limitations of the small cohort into account, paracetamol rescue medication was associated with less oversedation, suggesting safe postoperative analgesia in this population. Other quality care indicators, such as length of invasive mechanical ventilation or length of hospital stay and long-term outcome according to Bayley II developmental indexes, IVH, PLV, and CLD were similar in both the paracetamol (P) and non-paracetamol (non-P) groups of patients. Additionally, a detailed multimodal analysis focused on identifying deficiencies in pre-existing local postoperative pain management in the neonatal intensive care unit.

According to COMFORTneo, adequate pain control was only partially achieved in patients treated with paracetamol. On the other hand, the non-paracetamol group showed oversedation together along with more severe hypotension. Interestingly, the non-P group was significantly younger at the time of the surgery (median of PNA 8 vs. 29 days, respectively) and more vulnerable as speculated. Based on a local unit protocol, the rescue dose (RD) of intravenous paracetamol was administered to eleven neonates while in two patients, RD was added to analgesic drugs “routinely.” The median (IQR) COMFORTneo before the RD of paracetamol was 12 (9–13.3) within the target range but the decision to give the RD of paracetamol was based on the current situation when the neonate was considered as “uncomfortable” based on the standardized treatment protocol. The median (IQR) COMFORTneo after the RD was 13.9 (11–15), but the median (IQR) time to COMFORTneo reassessment was 5 (4–6) h after RD instead of 30–60 min as recommended in the literature because neonates were considered as “comfortable.” Moreover, no episode of obvious pain after the giving rescue paracetamol at the time of the COMFORT neo reassessment was reported by nurses. Recently published data on ELBW neonates treated for pain are in line with our results and support the need for improvement of neonatal pain management in ELBW neonates (16).

It seems the use of paracetamol in the “rescue” regimen was not significant in its effects to reduce the dose of opiates or the number of other analgesic boluses administered during the study period. The possible explanations may be (1) lack of consistency of caregivers in reassessing pain scores after interventions, as the daily number of assessments was the same between groups; (2) age-related differences in opiate pharmacokinetics; and (3) possible differences in interindividual disease characteristics and developmental changes in pain perception (23). Krekels et al. presented relevant data on a population pharmacokinetic (PK) model for morphine in (pre)term neonates. In their analysis, a similar difference in rescue medication and likely morphine over-exposure was observed in neonates with PNA<10 days. By reducing 50–75% of the routine 10 μg/kg/h infusion rate, steady-state concentrations of morphine and its metabolites were achieved. On contrary, in neonates ≥10 days of postnatal age (PNA), the infusion rate derived from PK modeling was higher than at the traditional dose (24, 25). Age-related changes in PK (e.g., greater distribution volume, lower clearance, higher free fraction of the drug in neonates) have also been known for synthetic opioids such as sufentanil (18, 26, 27). Drug clearance is generally not only driven by maturation but also by non-maturational covariates (e.g., disease-related differences in distribution and drug elimination) (28).

Opiates have been widely used analgesic agents in neonatal intensive care units in the past few decades despite negative short-term side effects and possible long-term neurobehavioral consequences for premature individuals (29–32). In contrast, the information on paracetamol is still more limited, for example, intravenous paracetamol is effective in reducing opioid consumption in term neonates and infants (15, 33, 34). However, there is limited evidence in ELBW neonates, in whom paracetamol is still off-label, for pain, or to treat patent ductus arteriosus (35). Recent studies show that the introduction of intravenous paracetamol as part of a postoperative pain management protocol along with the education of care providers leads to improved quality of care indicators even in premature infants. (e.g., reduction of analgesic and sedative consumption, shortening of mechanical ventilation, and parenteral nutrition) (36–39). Although these studies did not mention the usage of a loading dose of paracetamol. In our limited study population, the use of paracetamol to reduce the opioid dose was ineffective. This was probably due to inappropriate dosing and rescue analgesic medication adjustment which are unlikely to reach steady-state paracetamol concentration (40).

This study was performed to evaluate a standardized approach to postoperative pain (≤48 h) to achieve postoperative analgesic efficacy and safety objectives in ELBW neonates and meant as a baseline study for internal prospective validation of postoperative analgesia conducted in this population. The limitations of this study were, for example, the design of a retrospective observational study, the small sample size, and the initial phase of scoring implementation without an adequate reliability score among the caregivers' evidence. Another limiting factor of a designed postoperative follow-up period could be the amount of medication taken before and during surgery and the possible tolerance to opiates, especially in postnatally older patients. However, the median days of continuous opiate use preoperatively and their dosing did not statistically differ between the two study groups.

Therefore, implementing appropriate age-related dosing of opioids co-administered with intravenous paracetamol, including a loading dose, and setting up an educational program to achieve the best consistency and inter-rater reliability of healthcare professionals in pain assessment methods are the main goals of the prospective study. In addition, supporting parental contributions should be part of daily clinical practice. These interventions are the future direction of our project.

## Conclusion

The introduction of intravenous paracetamol as a rescue medication in ELBW neonates after abdominal surgery was safe analgesia, although it did not reduce opiate consumption in the rescue regimen. Prospective validation is needed to optimize postoperative analgesia according to analgesic efficacy and safety goals in this population.

## Data Availability Statement

The original contributions presented in the study are included in the article/Supplementary Material, further inquiries can be directed to the corresponding author.

## Ethics Statement

The studies involving human participants were reviewed and approved by Ethics Committee of General University Hospital in Prague (available at https://www.vfn.cz › odbornici › eticka-komise › informace). Written informed consent to participate in this study was provided by the participants' legal guardian/next of kin.

## Author Contributions

HC contributed to conceptualization, data curation, investigation, methodology, and writing—original draft. LB contributed to formal analysis and visualization. BZ contributed to investigation and resources. KA contributed to supervision and writing—original draft. PP contributed to conceptualization, methodology, project administration, supervision, and writing—original draft. All authors contributed to the article and approved the submitted version.

## Funding

This work was supported by the Ministry of Health of the Czech Republic [RVO VFN 64165]; and Charles University [UNCE 204064]. PP was supported by an unrestricted research grant of the Intensive Care of the Erasmus MC-Sophia Children's Hospital and Progress Q25.

## Conflict of Interest

The authors declare that the research was conducted in the absence of any commercial or financial relationships that could be construed as a potential conflict of interest.

## Publisher's Note

All claims expressed in this article are solely those of the authors and do not necessarily represent those of their affiliated organizations, or those of the publisher, the editors and the reviewers. Any product that may be evaluated in this article, or claim that may be made by its manufacturer, is not guaranteed or endorsed by the publisher.

## Abbreviations

BW: birth weight
BSID: Bayley Scales of Infant Development-BSID-II-mental (MDI) and psychomotor (PDI) developmental index
CLD: chronic lung disease
COMFORTneo: COMFORTneo scale
ELBW: extremely low birth weight
GA: gestational age
IVH: intraventricular hemorrhage
LD: loading dose
MD: maintenance dose
NEC: necrotizing enterocolitis
NICU: neonatal intensive care unit
Non-P group: non-paracetamol rescue group
NRS: numeric rating scale
P group: paracetamol rescue group
PD: pharmacodynamics
PK: pharmacokinetics
PMA: postmenstrual age
PNA: postnatal age
PVL: periventricular leukomalacia
RD: rescue dose
SIP: spontaneous intestinal perforation.
