# Supplementary material for: Rescue Paracetamol in Postoperative Pain Management in Extremely Low Birth Weight Neonates Following Abdominal Surgery: A Single Unit Retrospective Study
Source: Front Pediatr. 2022 Jun 23;10:895040. doi: 10.3389/fped.2022.895040 (PMC9262101; doi:10.3389/fped.2022.895040)
Supplement: Supplementary file 1 [file Data_Sheet_1.PDF]

*Supplementary Material*

# Rescue paracetamol in postoperative pain management in extremely low birth weight neonates following abdominal surgery: a single unit audit.

**Hana Cihlarova[1)], Lenka Bencova[2)], Blanka Zlatohlavkova[1)], Karel Allegaert[3,4)], Pavla Pokorna[2,3,5,6)]**

**Correspondence:**
Pokorna Pavla, MD, PhD
**pokornakarlov@seznam.cz**

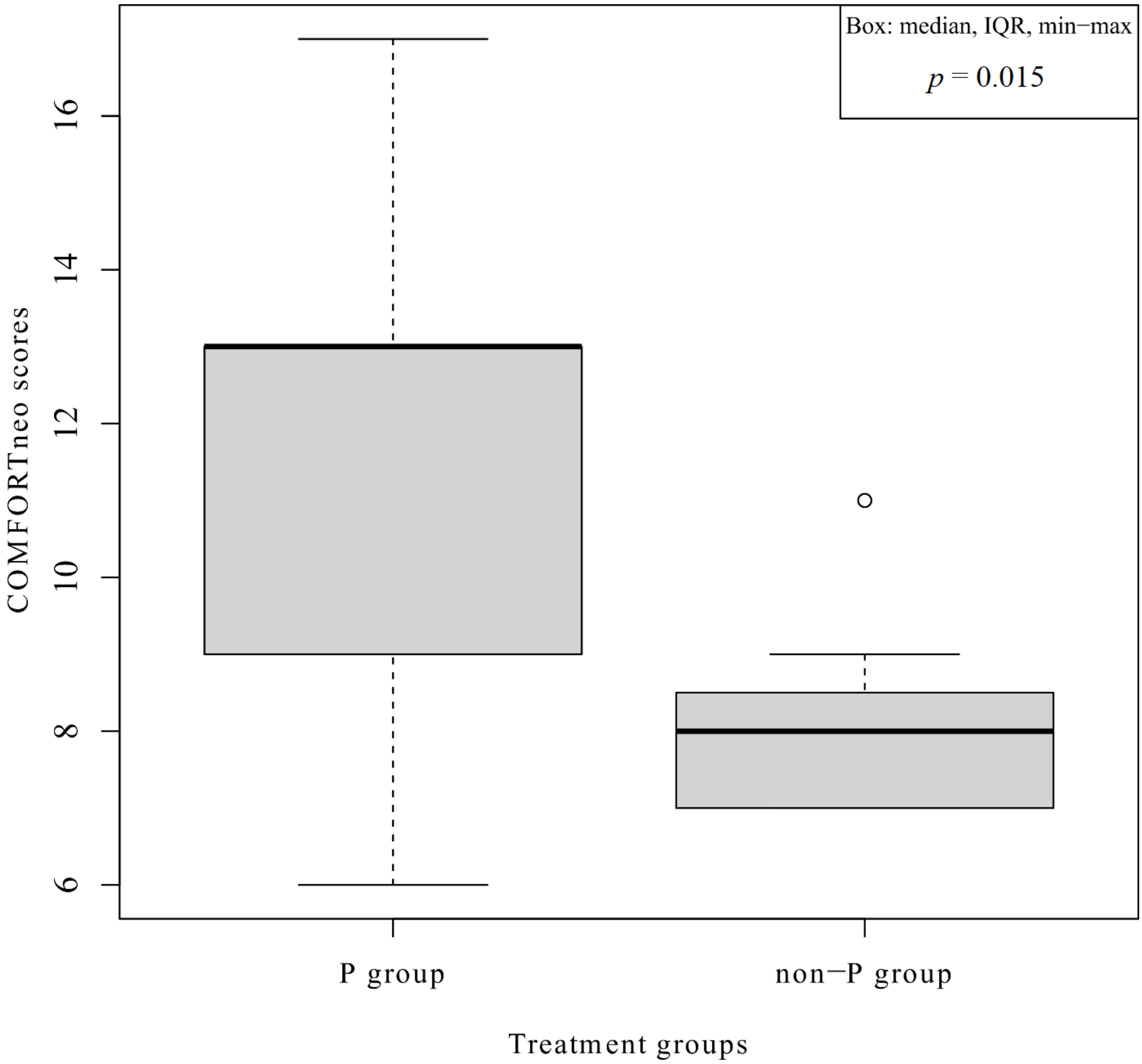


**Supplementary Figure 1:** Average COMFORTneo scores in paracetamol (P) and non-paracetamol (non-P) group over the first 24 hours postoperatively.

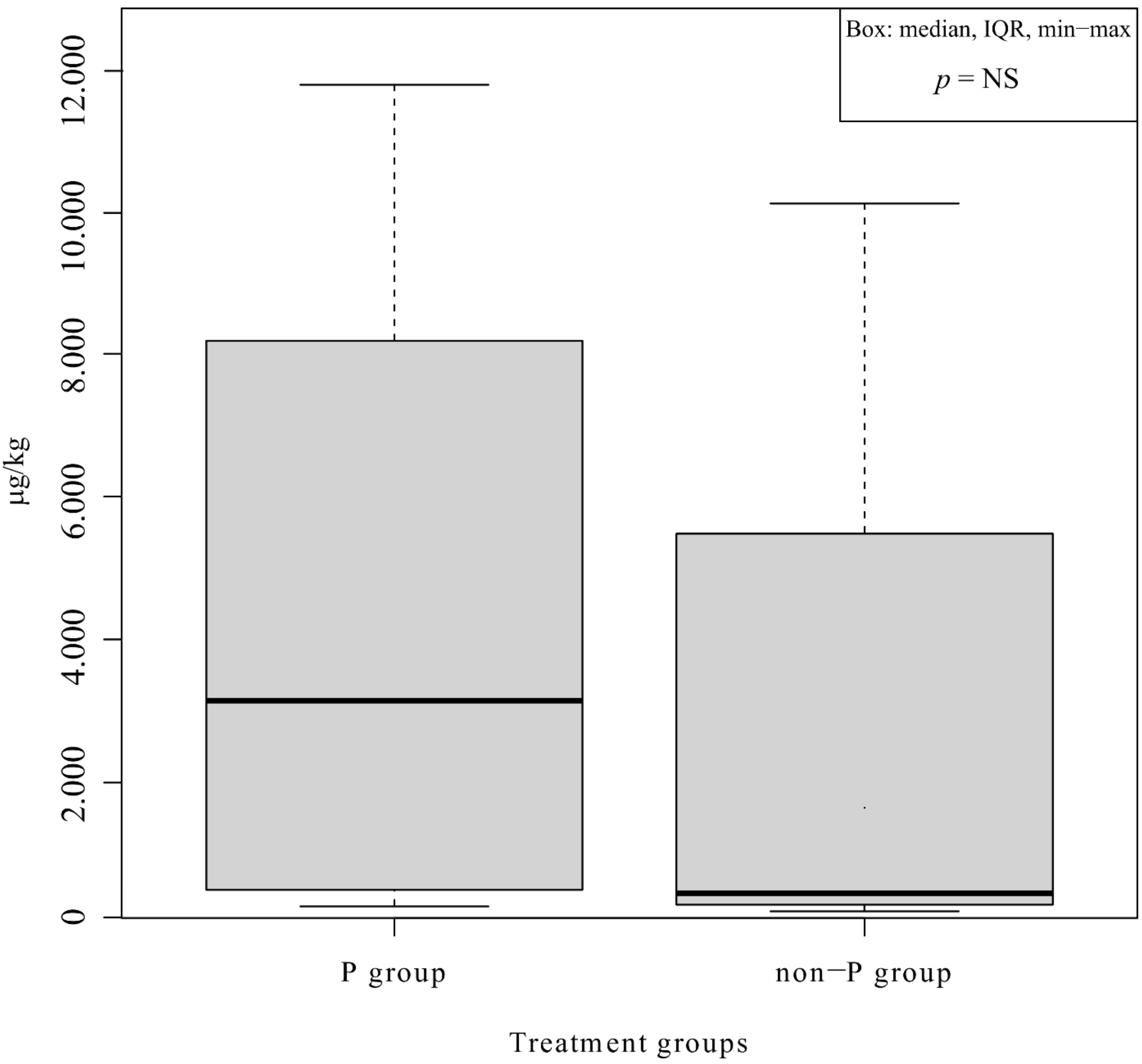


**Supplementary Figure 2:** Equivalent average daily dose of opiates - average daily dose of morphine and morphine equivalents (µg/kg) in paracetamol (P) and non-paracetamol (non-P) group is not significantly different (NS).
